# Synergistic Effects of Protein Intake and Exercise on Biomarkers of Sarcopenia: A Systematic Review

**DOI:** 10.3390/biom16020195

**Published:** 2026-01-27

**Authors:** Stephanie Cruz-Pierard, Samuel Iñiguez-Jiménez

**Affiliations:** 1 Carrera de Enfermería, Facultad de Ciencias de la Salud y Bienestar Humano, Universidad Tecnológica Indoamérica, Quito 170103, Ecuador; scruz586@puce.edu.ec; 2 Facultad de Salud y Bienestar, Pontificia Universidad Católica del Ecuador, Quito 170525, Ecuador; 3 Centro de Investigación para la Salud en América Latina (CISeAL), Quito 170530, Ecuador; 4 Escuela de Fisioterapia, Facultad de Ciencias Médicas, de la Salud y la Vida, Universidad Internacional del Ecuador, Quito 170105, Ecuador

**Keywords:** biomarkers, exercise, protein intake, sarcopenia

## Abstract

Sarcopenia, defined as the progressive decline of muscle mass, strength, and function, severely compromises autonomy and quality of life in older adults. This systematic review evaluated synergistic effects of protein supplementation combined with resistance exercise on biochemical and functional biomarkers of sarcopenia. The search for scientific evidence was conducted in PubMed, Scopus, ScienceDirect, and Cochrane databases (2019–2025), applying explicit inclusion and exclusion criteria, like only randomized controlled trials in humans, published in English, Spanish, or French, were included to ensure high-quality evidence. After selection, the risk of bias of the articles was assessed according to the Cochrane Handbook for Systematic Reviews of Interventions. Seven randomized controlled trials, with a total of 260 participants, met the eligibility criteria. Interventions combining resistance exercise three times per week at 60–80% of one-repetition maximum with daily protein supplementation of at least 15 g, mainly from dairy sources, showed synergistic effects. Improvements were observed in inflammatory and anabolic biomarkers, with reductions in myostatin, activin, and IL-6, and increases in IGF-1, follistatin, and IL-10. Functional outcomes included gains in muscle strength, fat-free mass, and muscle fiber cross-sectional area. Despite heterogeneity in duration and sample size, findings support this combined approach as a promising and clinically applicable strategy to prevent and treat sarcopenia. No external funding was received, and the review is registered in PROSPERO (CRD42025640989).

## 1. Introduction

Sarcopenia (Sp) is a public health condition of global significance, as it entails several complications that increase morbidity and mortality, particularly in older adults and hospitalized patients [1,2]. Currently, the global prevalence of sarcopenia ranges from 10% to 27% [3]. In Spain, a 2016 study including 276 participants (mean age 87.2 years, predominantly women) found that 37% had sarcopenia with reduced muscle mass, 86% exhibited slow gait, and 95% suffered from muscle weakness [4]. Sarcopenia’s prevalence increased with age, and 90% of participants also showed declines in strength and walking speed [4,5].

**Figure 1:**
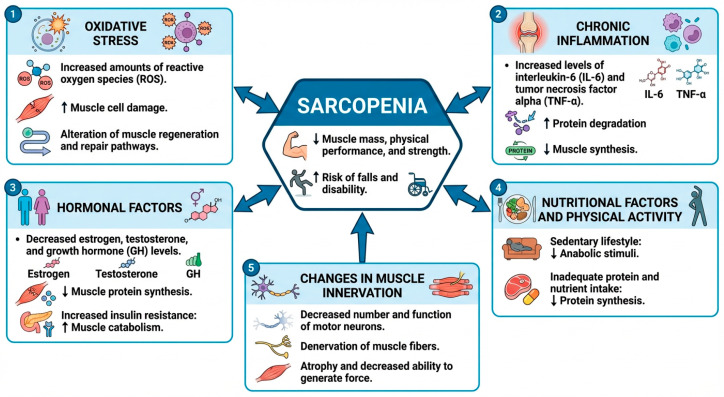
Pathophysiological mechanisms of sarcopenia.

The term sarcopenia derives from the Greek words *sarx* (flesh) and *penia* (loss/poverty) [6]. This condition is characterized by decreases in muscle mass, strength, and functionality in older adults [1,7]. Such muscle wasting and weakness can elevate the risk of falls, fractures, and other injuries that compromise an individual’s ability to perform activities of daily living [2,7]. Sarcopenia is a multifactorial condition whose pathophysiology involves aging, reduced physical activity, inadequate nutritional intake, and the presence of chronic diseases such as cancer [2,7]. As illustrated in Figure 1, its progression is governed by the interaction of inflammatory, endocrine, oxidative, and neuromuscular mechanisms.

Key alterations include increased levels of pro-inflammatory cytokines such as interleukin-6 and tumor necrosis factor-α, decreases in anabolic hormones including estrogen, testosterone, and growth hormone, excessive production of reactive oxygen species, and progressive impairment of neuromuscular innervation. Together, these alterations accelerate muscle protein breakdown, suppress protein synthesis, reduce anabolic signaling, and ultimately contribute to muscle catabolism and impaired regenerative capacity (Figure 1) [1,6].

For its diagnosis, according to the European Working Group on Sarcopenia in Older People (EWGSOP), sarcopenia is a skeletal muscle disease involving both muscle mass and strength [6]. However, loss of these components can occur separately, with specific terms for each: (A) kratopenia, referring to loss of power; (B) myopenia, referring to decreased muscle mass; and (C) dynapenia, referring to reduced strength [6]. Thus, sarcopenia per se represents the combination of kratopenia and myopenia, while severe sarcopenia also includes dynapenia [6]. According to the updated EWGSOP consensus, sarcopenia is considered present when decreased strength is detected (criterion 1). Diagnosis is confirmed by low muscle mass (criterion 2) and is classified as severe if there is also reduced physical performance or functionality (criterion 3) [6].

Screening for sarcopenia in primary care and geriatric centers can be conducted using practical and accessible methods, such as anthropometric measurements, bioelectrical impedance, or handgrip strength assessment [8]. At present, biochemical biomarkers play an increasingly important role not only in disease diagnosis but also in the monitoring of disease progression and therapeutic response. In sarcopenia, these biomarkers are classified into two groups: (A) those related to musculoskeletal status, such as myokines (myostatin, follistatin), muscle remodeling markers (N-terminal propeptide of type III collagen [PIIINP] and sarcopenia index [SI]); and (B) those analyzing causal factors, including metabolic, hormonal, and inflammatory markers such as insulin-like growth factor-1 (IGF-1), dehydroepiandrosterone sulfate (DHEA-S), cortisol, C-reactive protein (CRP), IL-6, and tumor necrosis factor-alpha (TNF-α) [9]. These biomarkers show the imbalance between catabolic and anabolic signaling pathways of sarcopenia at the molecular level [6,9]. Table 1 summarizes some of the most commonly used biochemical biomarkers for the diagnosis of sarcopenia.

One advantage of sarcopenia biomarkers is that they allow earlier diagnosis and timely treatment to prevent subsequent complications. Several studies have shown associations between sarcopenia and alterations in biological markers. For example, in a meta-analysis by Silva-Fohn et al. [10], older adults with sarcopenia had significantly lower blood albumin levels compared to healthy participants. Another study hypothesized that aging is associated with subclinical inflammation, characterized by increased IL-6 levels and decreased IGF-1 [11]. These changes contribute to accelerated muscle mass loss, as IGF-1 plays a crucial anabolic role in its maintenance [11].

Early and accurate diagnosis is highly important, as it helps prevent comorbidities that can severely affect health. Sarcopenia significantly impairs functionality in older adults, causing difficulties in walking, rising from a chair, or climbing stairs due to lower limb muscle weakness, which increases the risk of falls and, consequently, dependence, institutionalization, and mortality from related accidents [12]. Moreover, sarcopenia is associated with a higher risk of chronic diseases, such as osteoporosis and diabetes, since muscle mass loss impacts bone density and glucose tolerance [12].

Preventive measures and treatment options for this condition include diet quality, particularly protein intake, and regular physical activity, preferably muscle-strengthening exercises. In a study on the importance of nutritional management in older adults with sarcopenia, it was established that this condition can affect both undernourished and obese individuals. Recommended nutritional treatment includes a protein-rich diet, with an intake of 1–1.6 g/kg/day, combined with regular physical exercise [13].

Aging is commonly associated with an imbalance in protein intake, characterized by insufficient total consumption and a reduced anabolic response, a phenomenon known as anabolic resistance [5,13]. Under these conditions, the capacity to stimulate muscle protein synthesis is blunted even when protein is consumed, thereby favoring the progressive loss of muscle mass in older adults [1,6]. Inadequate protein intake is also linked to a state of chronic low-grade inflammation, evidenced by elevated interleukin-6 (IL-6) levels and diminished anti-inflammatory activity of interleukin-10 (IL-10), which together create a catabolic muscle environment [1,6,11].

At the molecular level, this inflammatory imbalance promotes the upregulation of myostatin and activin, potent inhibitors of muscle growth, while concurrently suppressing key anabolic mediators such as insulin-like growth factor-1 (IGF-1) and follistatin. These alterations impair satellite cell activation and reduce muscle protein synthesis [9,11]. As a result, insufficient protein intake not only limits the supply of essential amino acids required for muscle remodeling but also exacerbates inflammatory and catabolic signaling pathways that accelerate the progression of sarcopenia in the aging population [1,5,6,10,13].

Protein supplementation provides the essential amino acids necessary to support muscle protein synthesis, whereas resistance exercise enhances neuromuscular activation and increases muscle sensitivity to anabolic stimuli, producing a synergistic effect on muscle remodeling in older adults with sarcopenia [1,5,6,13]. Although a growing number of studies have examined the effects of exercise or protein intake independently, systematic evidence specifically addressing their combined impact on biochemical biomarkers of sarcopenia remains limited. In particular, the interaction between protein supplementation and resistance exercise on key anabolic (IGF-1, follistatin), catabolic (myostatin), and inflammatory (IL-6, IL-10) mediators has yet to be comprehensively synthesized [3,5,9,11].

Therefore, the present systematic review aims to establish the synergistic effects of protein intake and exercise on sarcopenia biomarkers, with the goal of improving patient quality of life and preventing complications that arise in the absence of appropriate treatment. By integrating molecular, metabolic, and functional outcomes, this review aims to provide both mechanistic insight and clinical support for the development of evidence-based strategies for the prevention and management of sarcopenia [1,3,5,6,12,13].

## 2. Materials and Methods

### 2.1. Protocol

A systematic review of the available literature was conducted following the Preferred Reporting Items for Systematic Reviews and Meta-Analyses (PRISMA) (Supplementary Materials) methodology [14]. The study was registered in the International Prospective Register for Systematic Reviews (PROSPERO), ID: CRD42025640989.

### 2.2. Search Strategy

Four search strategies were designed using MeSH terms, specifically adapted for the PubMed, Scopus, ScienceDirect, and Cochrane databases (Appendix A). The following filters were applied: randomized controlled trials, full-text availability, human studies, and publications in English, French, or Spanish; additionally, publications from the last five years were considered. In Scopus and ScienceDirect, the selected filters included language (French, English, and Spanish), document type (articles), human studies, and publication period (2019–2025). Cochrane included trials published between 2019 and 2025. To collect the most current evidence and compare results with previous reviews, analyzing potential variations in findings, the most recent five years were selected.

A total of 287 articles were identified in Cochrane, 106 in Scopus, 64 in ScienceDirect, and 72 in PubMed as potentially relevant. The most recent search was conducted on 6 March 2025.

### 2.3. Selection Criteria

Studies were included if they involved participants aged 65 years or older with sarcopenia or at risk of developing it and analyzed combined interventions of physical activity and protein intake, specifying the amount and type, as well as biomarkers of muscle function or sarcopenia. Only randomized controlled trials published in English, French, or Spanish within the previous five years were considered. Studies involving patients with conditions such as diabetes, cancer, obesity, liver or kidney disease, mobility issues, or trauma were excluded. Animal or in vitro studies, studies with a high risk of bias, and secondary data analyses were also excluded.

To minimize clinical heterogeneity and better isolate the specific effects of protein supplementation combined with resistance exercise on sarcopenia-related biomarkers, studies that primarily included participants with advanced chronic conditions, such as type 2 diabetes, obesity, overt cardiovascular disease, or active cancer, were excluded. These conditions are commonly associated with substantial metabolic and inflammatory disturbances that can independently influence muscle mass, function, and biomarker profiles, thereby complicating the attribution of observed effects to the interventions themselves. Nevertheless, it is acknowledged that this methodological decision restricts the direct generalizability of the findings to the broader older adult population, in which such comorbidities are highly prevalent.

### 2.4. Methods to Minimize Risk of Bias

The search for scientific evidence was conducted by applying inclusion and exclusion criteria. After selection, the risk of bias of the articles was assessed according to the Cochrane Handbook for Systematic Reviews of Interventions [15].

### 2.5. Data Extraction Method

**Figure 2:**
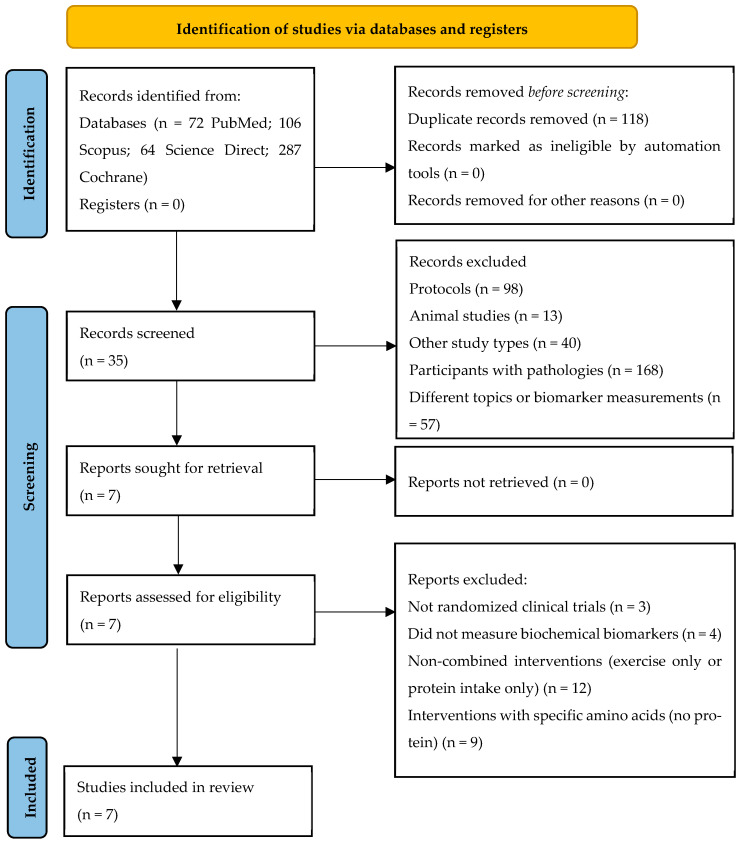
Item selection diagram.

Two reviewers independently performed the screening of titles and abstracts and the selection of eligible studies using Rayyan (online web version; Qatar Computing Research Institute, Doha, Qatar). The selection of articles is detailed in Figure 2. Then, a standardized form was employed to collect information on authors, year of publication, number and type of participants, intervention, biochemical biomarkers analyzed, outcomes, and conclusions (Table 2). Discrepancies were resolved through discussion and consensus.

### 2.6. Use of GenAI Tools

In the development of this publication, Generative AI (GenAI) techniques were utilized in compliance with MDPI’s principles for the responsible application of artificial intelligence. ChatGPT (OpenAI, GPT-5, September 2025 version) was utilized for grammatical refinement, enhancement of sentence structure, rectification of spelling and punctuation, and assistance in the organization and intelligibility of the content. Furthermore, the tool was used to assist in the drafting and optimization of search strategies by suggesting standardized terms and improving the wording of search strings. Additionally, AI-based image generation tools, including Nano Banana AI, were employed purely to create illustrative figures and graphical representations based entirely on notions set by the authors. These AI-generated images were just used for visualization purposes and did not contribute to data analysis, result interpretation, scientific originality, or text conceptual development. The authors personally reviewed, revised, and confirmed all AI-generated ideas to ensure correctness and scientific integrity.

## 3. Results

A total of seven randomized controlled trials were included, comprising 260 older adults. The main characteristics and outcomes of each study are summarized in Table 2. Intervention durations ranged from 8 weeks to 1 year and generally involved resistance training performed 2–3 times per week at intensities of 60–80% of one-repetition maximum, combined with a daily protein supplementation of at least 15 g, most frequently derived from dairy-based sources. Despite variability in study protocols, several trials reported simultaneous improvements in inflammatory and anabolic biomarkers alongside increases in muscle strength and fat-free mass. In contrast, other studies, including that of Bulow et al. [17], did not observe additional benefits of protein supplementation when combined with resistance training. The main findings have been divided into three main sections, along with other outcomes:

### 3.1. Protein Intake

Various nutritional interventions based on protein consumption, primarily from dairy sources, were evaluated [16,17,19,20,22]. Different protein sources (whey, collagen) were used in varying amounts (15 g, 20 g, 30 g, 45 g) and in combination with other nutrients (sucrose, leucine). The results suggest that a minimum intake of 15 g of complete protein from dairy sources, combined with exercise, may be beneficial in mitigating sarcopenia in older adults.

### 3.2. Role of Exercise

Various resistance training programs have been presented, each with specific characteristics regarding frequency (F), intensity (I), time (T), and type of exercise (TE). The common goal of the analyzed studies was to improve muscle strength and, potentially, some sarcopenia biomarkers.

This evidence demonstrates a positive effect on multiple biomarkers when strength-endurance training programs incorporate specific components like a frequency of three sessions per week, intensity at 60–80% of one-repetition maximum (1RM), volume of 2–4 sets of 8–12 repetitions per exercise, and compound movements targeting major muscle groups, performed for a minimum of eight weeks, with longer interventions also showing benefits [16,18,19,20,21,22]. In contrast, light-intensity resistance training does not appear to modify muscle protein synthesis [17].

### 3.3. Sarcopenia Biomarkers

The biomarker results suggest that the combination of exercise and protein supplementation in adults positively influences muscle regulation. A significant reduction in inflammatory biomarkers (IL-6, TNF-α, TGF-β, GDF15, and Activin A) [16,19,20] and an increase in IL-10 [20] were observed, suggesting a decrease in systemic inflammation associated with aging [16,19,20]. In parallel, an increase in IGF-1 and follistatin levels and a decrease in myostatin were identified, thus favoring the activation of anabolic processes and increasing myofibrillar protein synthesis [18,19,21,22].

On the other hand, a study with contradictory results was also found, since it determined that daily supplementation with protein (whey or collagen) or carbohydrates, with or without exercise, for one year, did not modify muscle protein synthesis or the metabolome in healthy older adults [17].

Overall, the available evidence indicates that the combination of resistance exercise and adequate protein intake is generally associated with favorable changes in inflammatory and anabolic biomarkers, as well as with increases in muscle mass and strength. Nevertheless, substantial heterogeneity in intervention duration, protein source, training intensity, and baseline health status limits direct comparability across studies. Moreover, the absence of synergistic effects in some trials underscores the need to better define optimal dose–response relationships for both dietary protein and resistance exercise in older adults with, or at risk of, sarcopenia.

### 3.4. Other Relevant Results

The findings in the analyzed studies highlight the importance of exercise and diet as a preventive and/or optimal treatment strategy for older adults with sarcopenia. In this regard, some relevant results are described below:

#### 3.4.1. Increase in Fat-Free Mass

A generalized increase in fat-free mass was observed, ranging from 0.49 kg to 1.3 kg, depending on the study group and protocol used [16,18,19,20].

#### 3.4.2. Increase in Lean Mass

A specific study [19] quantified the increase in lean mass at 1.3 kg for the diet intervention group and 0.6 kg for the exercise intervention group, reinforcing the idea that both factors contribute to the increase in muscle mass.

#### 3.4.3. Decreased Fat Mass

It has been shown that fat mass tends to decrease in the intervention groups, suggesting a positive effect of exercise and high biological value protein supplementation on body composition [16,20].

#### 3.4.4. Increased Muscle Volume

Some studies reported a significant increase in the cross-sectional area of type 1 and 2a myofibers, indicating muscle growth at the cellular level related to protein interventions and resistance exercise [18,20,21].

#### 3.4.5. Improvements in Strength

Several authors agree with pointing out significant improvements in muscle strength in their research, which is directly related to the increase in muscle mass and fiber cross-sectional area [18,20,21].

### 3.5. Risk of Bias Assessment

**Figure 3:**
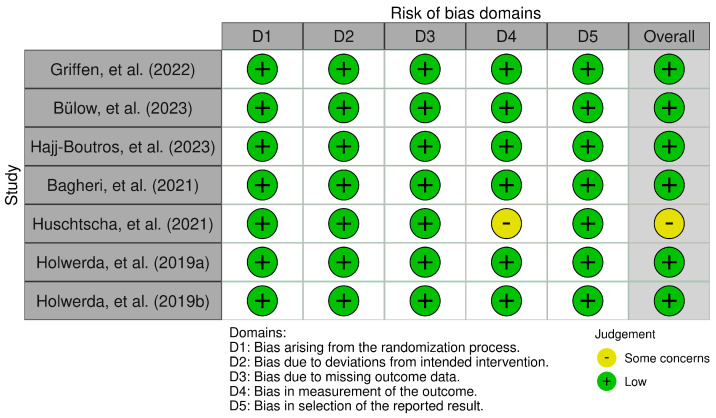
Risk of bias for each study analyzed [16,17,18,19,20,21,22].

**Figure 4:**
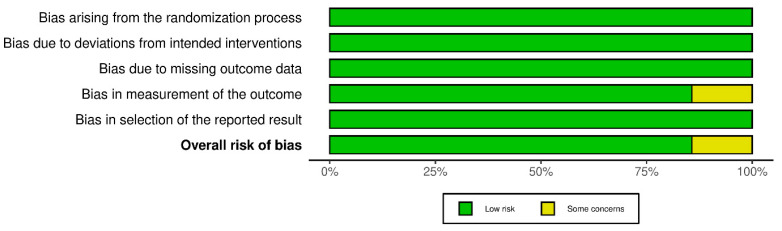
Overall risk of bias of the studies analyzed.

Finally, regarding the assessment of risk of bias in the analyzed studies, the Cochrane Risk of Bias 2.0 tool was applied. The corresponding summary graphs were generated using the robvis package (Version 0.3.0; University of Bristol, Bristol, UK). First, Figure 3 highlights that most analyzed studies present a low risk of bias [16,17,18,19,21,22]; only the study by Huschtscha et al. [20] showed a medium risk of bias in terms of attrition bias, since a large part of its population withdrew before completing the study for multiple reasons. On the other hand, Figure 4 summarizes the overall risk of bias distributed across the different domains evaluated, which showed a 12.5% risk of bias in the analyzed articles.

These methodological limitations may have weakened or masked potential synergistic effects of protein supplementation and resistance exercise on the evaluated biomarkers. Therefore, such sources of bias should be carefully considered when interpreting the overall strength and consistency of the available evidence.

## 4. Discussion

First, it is important to mention the various biochemical biomarkers used to assess sarcopenia. Although these are not yet widely used, increasing evidence supports their application in several studies due to their advantages, such as ease of obtaining blood values and rapid results [23,24].

**Figure 5:**
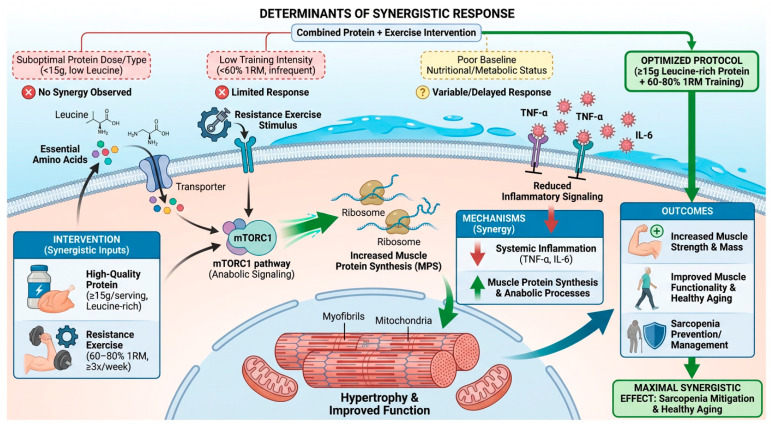
Determination of Synergistic Response.

In this review, inflammatory biomarkers such as interleukins (IL-6, TNF-α) were analyzed. Regarding sarcopenia, interleukins are associated with muscle mass loss and decreased strength, so the expected effect of exercise and protein intake is a reduction in these markers. This positive effect was observed in several studies included in this review [16,19,20] and confirmed in the meta-analysis by Nejati Bervanlou et al. [25], which reported significant reductions in IL-6 and TNF-α levels in intervention groups receiving exercise and protein (Figure 5). This effect may occur because whey proteins enhance cellular signaling and regulate the immune system, while exercise limits inflammasome activation and the release of inflammatory cytokines [26,27].

Conversely, IL-10 is a highly anti-inflammatory cytokine that plays a beneficial role in preserving muscle mass through mechanisms such as muscle regeneration and growth (promoting the transition of macrophages from the M1 to the M2 phenotype, which supports myoblast proliferation) [28]. In this context, the study by Huschtscha et al. [20] showed a significant increase in IL-10 in the combined intervention group (protein intake and exercise), partially consistent with the results of De Sá Souza et al. [29] and Pedraza-Vázquez et al. [30], who demonstrated that resistance exercise increases IL-10 levels, improving inflammatory parameters and physical performance in sarcopenic older adults. It can also modulate the expression of inflammation-related miRNAs, reduce the pro-inflammatory profile, and prevent muscle strength loss [30]. Additionally, Griffin et al. [16] reported that resistance exercise improved systemic inflammation without showing synergistic effects with protein supplementation.

Some reviewed studies analyzed growth factors (TGF-β, GDF15, activin, and IGF-1) and proteins regulating muscle mass, such as follistatin and myostatin, as sarcopenia biomarkers [19,20]. These biomarkers can be classified into two groups according to their effects on muscle mass: TGF-β, GDF15, activin, and myostatin negatively regulate muscle growth and induce atrophy (protein degradation, inhibition of protein synthesis, and suppression of muscle differentiation) [31], leading to reduced muscle strength [32]. In contrast, IGF-1 and follistatin are protective factors against muscle mass and strength loss [33,34]. IGF-1 is involved in protein synthesis and muscle growth, but its levels decrease with age, contributing to sarcopenia [33]. Follistatin counteracts the effects of myostatin and activins, inhibiting receptor activation and blocking pathways leading to muscle degradation [34]. It also promotes muscle fiber growth (protein synthesis) and improves metabolic function (insulin sensitivity) [35].

It is also important to note that baseline health status constitutes a potential confounding factor when interpreting the effects of protein supplementation on muscle-related outcomes. Older adults with conditions such as hyperlipidemia, type 2 diabetes, cardiovascular disease, or chronic kidney disease frequently exhibit metabolic alterations, chronic inflammation, and reduced or irregular physical activity patterns that may independently influence the risk of sarcopenia [2,5,6,12]. As most of the trials included in this review either excluded these conditions or did not provide a detailed characterization of them. The extent to which the observed effects can be generalized to multimorbid patients remains uncertain.

In this context, the interventions analyzed in this study, combining protein intake and exercise, showed positive effects, such as increased IGF-1 and follistatin levels, and decreased TGF-β, GDF15, and activin A [19]. These findings align with studies on protein consumption, exercise, and IGF-1 by Kazemi et al. [36] and Li et al. [37], which demonstrated that increased protein intake and resistance exercise elevate circulating IGF-1, promoting muscle anabolism. Follistatin levels also increased, and activin levels decreased with the consumption of 53 g of whey protein [38], while exercise independently increased follistatin [39]. No studies were found on the combined effects of protein and exercise on TGF-β and GDF15, indicating the need for further research.

Hormonal biomarkers of sarcopenia, such as insulin, cortisol, testosterone, and estradiol, were also analyzed [16,20] but showed no significant changes. This contrasts with other studies (Li et al. [40], Morley [41], Geraci et al. [42], Veronesi et al. [43]), where combined interventions of adequate protein intake and exercise positively influenced these hormonal biomarkers, improving muscle mass and function. This discrepancy may be explained by the short duration of interventions (3 months), participants’ healthy and active status, and other factors, including the use of hormonal replacement therapy in other studies showing positive results [42].

Another important aspect of sarcopenia biomarkers is muscle protein synthesis, which increased with protein intake and resistance exercise in several reviewed studies [18,21,22]. Myofibrillar protein synthesis (MyoPS) is essential for maintaining muscle mass, particularly in sarcopenia. Both adequate protein intake and resistance exercise promote MyoPS in older adults [44]. Evidence suggests that older adults require approximately 67% more daily protein to optimize postprandial muscle protein synthesis [44]. Protein quality and source are also important, with leucine-rich, high biological value proteins being most effective [21,44,45]. Resistance training further enhances MyoPS and preserves muscle mass, especially when combined with adequate protein intake [46,47].

Most studies identified synergistic positive effects of protein intake and exercise on sarcopenia biomarkers. However, this review also highlights the need to specify the type, amount, and characteristics of both macronutrients and exercise. Scientific literature recommends protein intake of 1.0–1.6 g/kg/day to maximize muscle protein synthesis and function [48]. High biological value proteins, such as whey, containing all essential amino acids, especially leucine, are considered most effective for stimulating protein synthesis [49]. Regarding exercise, personalized and regular resistance training improves both muscle mass and strength, enhancing protein synthesis in skeletal muscle cells [48]. The combination of these interventions can significantly improve sarcopenia-related signs and symptoms [49].

At the intracellular level, the synergistic effects of resistance exercise and protein intake on skeletal muscle can be attributed to the combined action of mechanical and nutritional stimuli on anabolic signaling pathways that regulate muscle protein synthesis (Figure 5). Resistance exercise enhances neuromuscular activation and amplifies intracellular signaling, thereby increasing muscle sensitivity to anabolic stimuli, while protein intake supplies the essential amino acids required to support muscle protein accretion [1,5,6,13]. Together, these stimuli promote the downregulation of myostatin, the upregulation of follistatin and insulin-like growth factor-1 (IGF-1), and the activation of satellite cells, ultimately favoring muscle hypertrophy and functional improvements in older adults with sarcopenia [9,11].

Other relevant findings included improvements in muscle strength, fat mass, lean mass, and muscle volume [16,18,19,20,21]. Viera et al. [50] reported that protein supplementation combined with resistance exercise plays a key role in counteracting age-related muscle loss, increasing lean mass, and increasing strength [50]. Other studies corroborate these effects, particularly with high biological value proteins, which increased muscle fiber cross-sectional area and volume when used with prolonged resistance training [51]. Protein supplementation effects on fat mass were less consistent [52], and some studies found no significant differences when adding protein to resistance training [52].

Another relevant aspect that warrants consideration is the role of overall dietary patterns and the contribution of other macronutrients. Total energy intake, lipid quality, and carbohydrate distribution can significantly influence body composition, metabolic health, and inflammatory status in older adults [1,5,6,13]. However, the trials included in this review did not report detailed information on total energy intake, fat and carbohydrate consumption, or changes in overall dietary composition throughout the intervention period. Consequently, it cannot be ruled out that unreported variations in energy balance or macronutrient distribution may have partially contributed to the observed changes in muscle mass, function, and biomarker profiles. Future studies should systematically monitor these nutritional variables in order to better disentangle the specific effects of protein supplementation from those of the overall dietary pattern.

In addition, none of the included trials provided comprehensive metabolic or hematological data, such as longitudinal body mass index trajectories, lipid profile parameters (triglycerides and total cholesterol), or indicators of cardiorespiratory fitness (VO_2_ max). As a result, potential interactions between protein intake, exercise-induced improvements in physical fitness, metabolic health, and sarcopenia-related biomarkers could not be fully explored. The incorporation of these outcomes in future randomized trials would allow a more complete understanding of whether improvements in muscle-related biomarkers are mediated, at least in part, by broader changes in metabolic and cardiovascular risk profiles.

When reduced protein intake or indicators of undernutrition are identified in older adults with, or at risk of, sarcopenia, it is essential to consider the underlying causes. These may include inadequate dietary intake, diminished appetite, socioeconomic constraints, the presence of acute or chronic catabolic diseases, and malabsorption, among other factors [1,5,10,13]. None of the trials included in this review systematically examined these determinants, which limits the ability to establish causal relationships between low protein intake, biomarker alterations, and clinical outcomes. Future interventions should therefore integrate quantitative nutritional assessment with an exploration of the determinants of malnutrition in order to design more targeted and sustainable therapeutic strategies.

Also, the potential influence of protein type and quality, baseline metabolic status, and sex-related differences on the response to combined protein supplementation and resistance exercise remains insufficiently characterized. Most of the included studies relied on dairy-based protein supplements and did not directly compare different protein sources or amino acid profiles. Addressing these aspects in future research would contribute to refining protein recommendations according to sex, metabolic status, and individual risk profiles.

Overall, these results suggest positive effects on sarcopenia indicators beyond the previously mentioned biomarkers, reinforcing the efficacy of these interventions in improving body composition and muscle function, thereby preventing sarcopenia. However, individual variability and factors such as age, nutritional status, and exercise intensity should be considered. In the other hand, the absence of a significant effect reported by Bülow et al. [17] may be partly explained by the relatively healthy and physically active baseline status of the participants, the extended duration of the intervention with the possibility of adaptive plateaus, and the lack of a clearly defined anabolic threshold for protein dosing. In addition, the use of collagen protein, characterized by a low content of essential amino acids and leucine, may have limited its anabolic potential when compared with whey-based supplementation strategies.

Although systemic hormones such as insulin and cortisol have not shown consistent changes after interventions combining protein intake with resistance training, emerging human studies indicate that skeletal muscle adapts mainly through local intracellular signaling rather than through broad endocrine fluctuations. Evidence shows that resistance exercise paired with whey-protein ingestion enhances myofibrillar protein synthesis via mTOR activation in muscle tissue, even when circulating hormone levels remain relatively stable [53,54]. Additionally, protein supplementation above the recommended dietary allowance reinforces anabolic signaling and helps overcome age-related anabolic resistance by sustaining mTORC1 and rpS6 activation, thereby promoting muscle protein accretion in older adults [54]. Taken together, these findings suggest that the improvements observed in anabolic biomarkers and muscle function in aging populations are better explained by muscle-specific signaling mechanisms than by systemic hormonal changes.

Finally, this systematic review has several strengths. It addresses the important topic of combined protein intake and exercise on sarcopenia biomarkers, using novel precision nutrition tools. Rigorous inclusion and exclusion criteria, along with validated methodologies such as PRISMA and Cochrane risk-of-bias assessment, ensure the quality and reliability of the results, supported by evidence from the last five years. 

### Limitations and Perspectives

The study also has limitations, such as the small number of analyzed studies, which limits generalizability and highlights the need for further research. There was considerable heterogeneity in study populations (age, health status, demographics) and interventions, complicating comparisons and the generation of specific conclusions.

An important limitation of the available evidence is that several trials, including those analyzed in this review, excluded older adults with clinically relevant comorbidities such as obesity, type 2 diabetes, dyslipidemia, or cardiovascular disease. While this approach enhances internal validity, it limits external applicability, as real-world older populations commonly present with multiple metabolic and cardiovascular conditions that may modify the response to combined protein and exercise interventions [2,5,6,12]. Therefore, caution is warranted when extrapolating these findings to sarcopenic patients with more complex health profiles.

Future studies should explore in more detail and over longer periods the effects of different exercise protocols and protein supplementation on biochemical sarcopenia parameters to optimize strategies for prevention and treatment.

## 5. Conclusions

Intake of high-quality protein (≥15 g per serving, particularly rich in leucine) combined with moderate to high-intensity resistance exercise (60–80% of one-repetition maximum, at least three times per week) exerts a synergistic beneficial effect on sarcopenia biomarkers in older adults. This combined intervention stimulates muscle protein synthesis, activates anabolic processes, increases muscle strength and mass, and reduces systemic inflammatory biomarkers such as TNF-α and IL-6. The findings highlight the potential of these strategies may be effective tools for the prevention and proper management of sarcopenia, supporting muscle functionality and promoting healthy aging with an improved quality of life.

Nevertheless, the lack of synergistic effects observed in some trials indicates that the magnitude of the response is highly dependent on specific intervention characteristics, including protein type and dosage, training intensity, and the participants’ baseline nutritional and metabolic status.

## Figures and Tables

**Table 1:** Types of biochemical biomarkers of sarcopenia.

Type	Biomarkers	Description and Relevance in Sarcopenia
Muscle	Myostatin, Creatine Kinase (CK), and Follistatin.	Indicators of muscle metabolism, muscle protein degradation, and inhibition of muscle growth. Myostatin inhibits muscle protein synthesis, while follistatin antagonizes myostatin and promotes hypertrophy [9].
Inflammatory	IL-6, TNF-α, CRP, and IL-10.	Pro-inflammatory cytokines promote protein degradation and inhibit muscle synthesis, contributing to chronic low-grade inflammation, while IL-10 exerts anti-inflammatory effects [1,6].
Endocrine	Growth Hormone (GH), Testosterone, Insulin, and IGF-1.	Endocrine factors that decline with aging are directly involved in anabolic signaling and preservation of muscle mass and strength, particularly IGF-1 [1,6].
Bone metabolism and serum proteins	Prealbumin, Collagen degradation proteins, Osteocalcin, and Albumin.	Indicators of bone and blood protein metabolism. Low albumin levels are associated with sarcopenia and poor prognosis in older adults [5,9,10].
Oxidative stress	Superoxide dismutase (SOD), Malondialdehyde (MDA), and Glutathione peroxidase (GPx).	Markers of oxidative muscle damage and imbalance in free radical production and elimination. Oxidative stress contributes to muscle fiber damage and impaired regeneration [4,6].
Nutritional	Levels of leucine or other amino acids, Vitamin D, and Omega-3 fatty acids.	Nutrients relevant to protein synthesis, muscle regeneration, and prevention of sarcopenia. Adequate protein and micronutrient intake is essential to counteract anabolic resistance in older adults [5,9].

**Table 2:** Results of the studies included in the review.

Author and Year of Publication	Number and Type of Participants	Intervention	Biochemical Biomarkers Evaluated	Results	Conclusions
Griffen et al. (2022) [16].	Healthy older men, mean age 67 ± 1 years (n = 36). Control group (n = 9). Whey protein group (n = 9). Exercise + control group (n = 9). Exercise + whey protein group (n = 9).	12-week intervention: Resistance exercise: Whole-body sessions, twice a week. 2 sets of 8 repetitions and 1 set to voluntary muscular failure at 80% of 1RM. Supplementation: 25 g of whey protein isolate, twice daily. Control: 23.75 g of maltodextrin, twice daily.	Hormonal: Unspecified. Inflammatory: IL-6 and TNF-α.	Systemic inflammation: Reduction in IL-6 (−1.0 ± 0.4 pg/mL) and TNF-α (−0.7 ± 0.3 pg/mL) in the exercise groups. Fat-free mass increased (+0.9 ± 0.3 kg), while fat mass decreased (−0.4 ± 0.4 kg).	Resistance exercise improved levels of systemic inflammation. Protein supplementation did not show synergistic effects with exercise.
Bülow et al. (2023) [17].	Healthy older adults, aged 65 years or older (n = 66, 29 women, 37 men). CHO group (n = 12). Collagen group (n = 15). Whey group (n = 15). Light resistance training group with whey (n = 12). Heavy resistance training group with whey (n = 12, 8).	12-month intervention: Carbohydrate supplementation (20 g of maltodextrin + 10 g of sucrose). Collagen supplementation (20 g of hydrolyzed bovine collagen protein + 10 g of sucrose). Whey supplementation (20 g of whey protein isolate + 10 g of sucrose). All were taken twice daily. Light resistance training at home with whey supplementation. Supervised heavy resistance training in a center with whey supplementation.	Muscle protein synthesis is measured by deuterium (D_2_O). Muscle metabolome: gas chromatography and GC–MS, at baseline and 4 h after consumption of a shake containing 20 g of hydrolyzed whey protein and 10 g of glucose.	No significant differences were observed between groups in the basal rate of muscle protein synthesis or in the postprandial response to protein supplementation after 12 months. Changes in basal and postprandial rates were minimal and similar between groups. Seventy-four major metabolites were identified by GC–MS, with no significant alterations in the muscle metabolomic profile before and after supplementation. However, 3-hydroxybutyric acid and 2-butenedioic acid consistently decreased by 4 h post-ingestion, indicating a possible relationship with postprandial energy metabolism.	Daily protein supplementation (whey or collagen), combined or not with carbohydrates and exercise, for one year did not induce significant changes in muscle protein synthesis or the metabolic profile of healthy older adults. These results suggest that, in this population, long-term interventions with these supplements may not generate relevant metabolic adaptations.
Hajj-Boutros et al. (2023) [18].	Pre-frail or frail older women, mean age 77.5 ± 1.3 years (n = 19) Intervention group: (n = 10). Placebo control group: (n = 9)	12-week intervention: Training: Progressive RT training program. Protein-optimized diet (1.2 g of protein/kg of body weight per day). Supplementation: 7.5 g/day of Leu. Placebo: Ala.	Myofibrillar fractional synthesis rate: in postabsorptive and postprandial states by infusion of L-[ring-2H5] phenylalanine.	Effect of resistance training with an optimized diet: Significant 66% increase in the basal rate of myofibrillar fractional synthesis. Effect of leucine supplementation: No additional benefit was observed with leucine supplementation compared to placebo.	The combination of resistance training and a high-protein diet significantly improved the frailty phenotype, increasing physical function, muscle strength, basal rate of muscle protein synthesis, and lean mass. However, additional leucine supplementation provided no additional benefits compared to placebo.
Bagheri et al. (2022) [19].	Healthy older adults, mean age 68 ± 4 years (n = 30). Intervention group (n = 15). Control group (n = 15).	Duration: 8 weeks. Experimental group: Training and consumption of 200 g of Icelandic yogurt (18 g of protein) after each session. Control group: Performed the same training but consumed a placebo (protein-free pudding). Training: Frequency: 3 times per week. Type: Strength training. Intensity: Moderate to high, 60–80% 1RM. Volume: 3 sets of 8–12 repetitions per exercise.	Muscle regulatory factors: IGF-1, TGF-β1, GDF15, Activin A, MST, and FST evaluated in serum using ELISA kits.	Positive changes were observed in several blood markers related to muscle growth and repair. For example, IGF-1 levels increased, and inflammatory markers such as TGF-β, GDF15, and activin A decreased. Furthermore, FST increased and MST decreased, suggesting greater activation of muscle growth processes and a significant improvement in strength.	Consumption of Icelandic yogurt after training significantly increased gains in lean muscle mass and strength compared to placebo. Icelandic yogurt may be an effective and cost-effective strategy to improve muscle health in older men and prevent sarcopenia.
Huschtscha et al. (2021) [20].	Healthy older adults (n = 37). DM (n = 8). EX + DM (n = 9). EX (n = 10). CON (n = 10).	12-week intervention: Groups: DM and EX + DM: Consumption of 500 mL/day of low-fat milk (15 g of protein per serving). EX and EX + DM: Supervised progressive resistance training three times per week. CON: Self-directed physical activity and diet without specific interventions.	Hormonal: Insulin, cortisol, IGF-1, testosterone, and estradiol. Inflammatory cytokines: IL-10, TNF-α, IL-6, IL-8, and IL-2.	Insulin levels: no significant changes in all groups. Cortisol levels: no changes in all groups. IGF-1, testosterone, and estradiol levels: no significant differences between groups. Cytokine IL-10: significant increase in the EX + DM group (88% at 6 weeks and 46% at 12 weeks). Absolute fat-free mass increased in the EX + DM groups. No significant changes in the remaining cytokines.	High-protein milk consumption combined with resistance training significantly improves muscle strength in active older adults. No additional effects on fat-free mass or hormonal biomarkers were observed compared to exercise alone.
Holwerda et al. (2019a) [21].	Older adults, mean age 67 ± 1 year (n = 24). 15 g protein group (n = 12). 15 g protein group with 1.5 g of free crystalline leucine (n = 12).	Both groups underwent a single 60 minutes bout of moderate-to-high-intensity whole-body resistance exercise. Supplementation included either 15 g of protein or 15 g of protein plus crystalline-free leucine.	Postmeal protein digestion kinetics and amino acid absorption and muscle protein synthesis rates were evaluated using continuous infusions of radiolabeled phenylalanine, tyrosine, and leucine.	Post-exercise myofibrillar protein synthesis rates were 16% higher in the 15 g + leucine group compared to the 15 g protein group. Similar results were obtained using L-[1-13C] leucine as a marker (19% higher).	Leucine co-ingestion further increases the post-exercise muscle protein synthetic response to the ingestion of a single 15 g dose of protein in older men.
Holwerda et al. (2019b) [22].	Healthy older adults, mean age 66 ± 1 years (n = 48). PLA group (n = 12). 15G group (n = 12). 30G group (n = 12). 45G group (n = 12).	32-week intervention: Different doses of milk protein (0, 15, 30, and 45 g) affected muscle protein synthesis after a resistance training session. Participants performed compound exercises at 75–80% of their 1RM, and muscle biopsies were taken before and 6 h after exercise. The milk protein contained labeled amino acids to measure protein synthesis.	Myofibrillar protein synthesis. Net body protein balance. Incorporation of amino acids derived from dietary protein into muscle. Plasma concentrations of essential amino acids and isotopic tracers.	Dose-dependent increase in total body net protein balance after ingestion of 0, 15, 30, or 45 g of protein. Higher myofibrillar protein synthesis rates after ingestion of 30 or 45 g of protein. Incorporation of amino acids from dietary protein (phenylalanine) into de novo myofibrillar protein showed a dose-dependent increase after ingestion of 15, 30, or 45 g of protein.	Ingesting at least 30 g of protein after exercise promotes muscle protein synthesis, helping to counteract age-related anabolic resistance. Increasing dietary protein after exercise is considered a positive strategy for preventing sarcopenia in older populations.

1RM: one-repetition maximum. IL-6: Interleukin-6. TNF-α: Tumor necrosis factor alpha. GC–MS: Mass spectrometry. RT: Resistance. Leu: Leucine. Ala: Alanine. IGF-1: Insulin-like growth factor 1. TGF-β1: Transforming growth factor beta 1. GDF15: Growth differentiation factor 15. MST: Myostatin. FST: Follistatin. ELISA: Enzyme-linked immunosorbent assay. DM: High protein milk consumption. EX + DM: Exercise + milk. EX: Exercise only. CON: Control group. IL-10: Interleukin 10. IL-8: Interleukin 8. IL-2: Interleukin 2. PLA: Placebo.

## Data Availability

No new data were created or analyzed in this study.

## Supplementary Materials

The following supporting information can be downloaded at: https://www.mdpi.com/article/10.3390/biom16020195/s1, PRISMA 2020 Checklist [55].

## Appendix A

**Table A1:** Search strings.

Database	Search Strategy
Pubmed	((“Sarcopenia”[Mesh] OR sarcopenia[tiab] OR “muscle wasting”[tiab] OR “muscle loss”[tiab] OR “muscle atrophy”[tiab]) AND (“Dietary Proteins”[Mesh] OR “Amino Acids”[Mesh] OR dietary protein [tiab] OR protein [tiab] OR “protein supplementation”[tiab] OR supplement [tiab] OR leucine[tiab] OR whey[tiab] OR “essential amino acid”[tiab]) AND (“Exercise”[Mesh] OR “Exercise Therapy”[Mesh] OR exercise[tiab] OR “resistance training”[tiab] OR “strength training”[tiab] OR “physical activity”[tiab]) AND (biomarker[Mesh] OR “muscle mass”[tiab] OR “lean mass”[tiab] OR “muscle strength”[tiab] OR “grip strength”[tiab] OR “gait speed”[tiab] OR “physical performance”[tiab] OR myostatin[tiab] OR IGF-1[tiab] OR interleukin-6[tiab] OR IL-6[tiab] OR CRP[tiab]))
Scopus	TITLE-ABS-KEY( sarcopenia OR “muscle wasting” OR “muscle loss” OR “muscle atrophy”) AND TITLE-ABS-KEY( protein OR “dietary protein” OR “protein supplementation” OR leucine OR whey OR “essential amino acid”) AND TITLE-ABS-KEY(exercise OR “resistance training” OR “strength training” OR “physical activity” OR “exercise therapy”) AND TITLE-ABS-KEY(biomarker OR “muscle mass” OR “lean mass” OR “muscle strength” OR “grip strength” OR “gait speed” OR “physical performance” OR myostatin OR “IGF-1” OR “interleukin 6” OR CRP )
ScienceDirect	(“sarcopenia” OR “muscle wasting” OR “muscle loss” OR “muscle atrophy”) AND (“protein” OR “dietary protein” OR “protein supplementation” OR “amino acid” OR “leucine” OR “whey”) AND (“exercise” OR “resistance training” OR “strength training” OR “physical activity” OR “exercise therapy”) AND (“biomarker” OR “muscle mass” OR “lean mass” OR “muscle strength” OR “grip strength” OR “gait speed” OR “physical performance” OR “myostatin” OR “IGF-1” OR “IL-6” OR “CRP”)
Cochrane	1 MeSH descriptor: [Sarcopenia] explode all trees, 2 sarcopenia OR “muscle wasting” OR “muscle loss” OR “muscle atrophy”, 3 MeSH descriptor: [Dietary Proteins] explode all trees, 4 protein* OR “dietary protein” OR “protein supple-mentation” OR leucine OR whey, 5 MeSH descriptor: [Exercise] explode all trees, 6 exercise OR “resistance training” OR “strength training” OR “physical activity” OR “exercise therapy”, 7 MeSH descriptor: [Biological Markers] explode all trees, 8 biomarker OR “muscle mass” OR “lean mass” OR “muscle strength” OR “grip strength” OR “gait speed” OR myostatin OR IGF-1 OR IL-6 OR CRP, Final: (1 OR 2) AND (3 OR 4) AND (5 OR 6) AND (7 OR 8)
